# Examination of Cluster Groups of Risk Behaviors and Beliefs Associated with Non-Communicable Diseases with Latent Class Analysis: A Cross-Sectional Study in Rural Bangladesh

**DOI:** 10.3390/healthcare11162279

**Published:** 2023-08-12

**Authors:** Yurie Kobashi, Syed Emdadul Haque, Isamu Amir, Kayako Sakisaka, Sanzida Mubassara, Masaharu Tsubokura

**Affiliations:** 1 Department of Radiation Health Management, Fukushima Medical University School of Medicine, Fukushima City 960-1295, Fukushima, Japan; iamir@fmu.ac.jp (I.A.); tsubo-m@fmu.ac.jp (M.T.); 2 Global Exchange Center, Fukushima Medical University School of Medicine, Fukushima City 960-1295, Fukjushima, Japan; 3 Health Equity Research Institute, Abiko City 270-1168, Chiba, Japan; 4 UChicago Research Bangladesh, Dhaka 1230, Bangladesh; emdad91@gmail.com; 5 Faculty of International Liberal Arts, Kaichi International University, Kashiwa-City 277-0005, Chiba, Japan; k.sakisaka@kaichi.ac.jp; 6 Department of Botany, Jahangirnagar University, Savar Union 1342, Bangladesh; sanzida.botany@gmail.com

**Keywords:** non-communicable diseases, community survey, community-based participatory research, global health, Bangladesh

## Abstract

This cross-sectional observational study examined the cluster groups of risk behaviors and beliefs associated with non-communicable diseases (NCDs) and the demographic factors that influence these cluster groups. The questionnaire survey was conducted in Lohagara Upazila in Narail District, Bangladesh and included basic demographics and items associated with NCDs. The inclusion criteria for the participants in this study included those who were aged between 20 and 80 years and both sexes. The survey items were based on risk behavior, belief, and improvement behavior. To identify the several cluster groups based on NCD-related behavior and belief patterns, a log-likelihood latent class analysis was conducted. Then, a multinomial regression analysis was performed to identify the factor associated with each cluster group. Of the 600 participants, 231 (38.5%) had hypertension, 87 (14.5%) had diabetes, and 209 (34.8%) had a body mass index of 25 or more. Finally, risk behaviors and beliefs associated with NCDs were classified into three cluster groups: (1) very high-risk group (*n* = 58); (2) high-risk group (*n* = 270); and (3) moderate-risk group (*n* = 272). The very high-risk group was significantly associated with female gender, older age, fewer years spent in education, and the absence of daily medication compared to the moderate-risk group. Educational interventions in rural Bangladesh should be immediately implemented to improve the risk behaviors and beliefs associated with NCDs.

## 1. Introduction

Non-communicable diseases (NCDs) are the leading cause of early mortality and disease burden worldwide. Approximately 80% of NCDs occur in low-income countries (LICs) and middle-income countries (MICs) [1,2]. Further, two-thirds of cardiovascular-disease-related deaths occur in LICs and MICs [3]. The causes of the high prevalence of NCDs in developing countries have unprecedented complexity. The risk factors of NCDs include low health literacy and adherence, prenatal malnutrition, air pollution, dyslipidemia, smoking habits, obesity, alcohol consumption, unhealthy diet, loss of physical activity, depression, behavioral factors, and poverty [1,3,4,5,6,7,8,9,10,11,12,13]. These can be classified into five categories: self-management, genetic, environmental, medical condition, and sociodemographic factors [14]. These categories might be classified into different levels including the individual level, social level, and environmental level. Further, the unmet needs for medical resources and the poverty in LICs and MICs make it difficult to provide adequate medical care to each patient. Thus, the use of preventive medicine is the key strategy in these countries. Therefore, identifying the risk behaviors associated with NCDs, which can be self-managed at the community level, is a vital public health issue in developing countries.

Various studies have been conducted in LICs and MICs to identify the risk behaviors associated with NCDs at the community level. Regional disparity is a crucial problem related to NCDs, and previous studies have reported that each disease and risk behavior differs regionally within a country [15,16]. While urban areas face problems such as air pollution and inadequate diet habits, rural areas experience different issues, such as poverty and poor access to medical resources [15,16,17,18]. Notably, healthcare resources are very limited in rural areas of developing countries. Hence, the strategy of region-specific preventive medicine to reduce self-manageable risk behaviors should be immediately adopted. However, region-specific information on risk behaviors and beliefs regarding NCDs related to the rural areas of developing countries is limited.

Bangladesh is a lower-middle-income country (LMIC) located in Southeast Asia [19]. In rural Bangladesh, NCDs are a major cause of mortality [20]. Notably, 77% of qualified healthcare workers are concentrated in the urban areas of Bangladesh, and hence, the availability of healthcare professionals in rural areas is very limited [21]. Moreover, the economic disparity between urban and rural residents is a major problem in Bangladesh. Approximately 25% of the rural population is in the lowest wealth quintile compared to 8% of the urban population [22]. Therefore, rural Bangladesh was a suitable area for the survey on risk behaviors and beliefs associated with NCDs in order to develop a prevention strategy to protect the rural residents from deaths caused by NCDs in the future.

We examined the cluster groups of risk behaviors and beliefs associated with NCDs and the factors that influence these cluster groups by conducting a latent class analysis cross-sectionally of the rural population of Bangladesh.

## 2. Materials and Methods

### 2.1. Study Design and Participants

This study used a cross-sectional observational design. It was conducted as part of the Narail NCDs community survey. This study was reviewed and approved by the Biosafety, Biosecurity, and Ethical Committee of Jahangirnagar University, Dhaka, Bangladesh (Number: BBEC, JU/M 2021(9)2), and the ethical committee of Fukushima Medical University, Japan (Number: 2020-286). Verbal informed consent was obtained from the respondents before the interview.

The field survey was conducted in Lohagara Upazila (the smallest administrative unit in Bangladesh) in Narail District (Figure S1). Lohagara Upazila has 12 unions, of which 6 (i.e., Chorkhali, Rajupur, Kochubaria, Erenda, Permollikpur, and Chachoy) were selected for the community survey. Lohagara Upazila has a total area of 284.91 square kilometers (110.00 sq mi) [23]. According to the 2011 Bangladesh census, Lohagara Upazila had 51,233 households and a population of 228,594, of which 11.1% lived in urban areas [24]. The number of targeted populations, aged 20-80, in Lohagara Upazila was 129,833, approximately 55% of the whole population [25].

The inclusion criteria for the participants in this study included those who were aged between 20 and 80 years, both sexes, and those who agreed to participate in the whole project of the Narail NCDs community survey.

### 2.2. Procedure

To identify the cluster of risk behaviors and beliefs associated with NCDs, we administered a questionnaire survey to 600 residents between 15 March and 12 July 2022. The survey was composed of basic demographics and items of risk factors of NCDs. The questionnaire was created by referring to the WHO STEPwise approach to NCD risk factor surveillance (STEPS), a popular survey questionnaire on NCDs [26]. To make the questionnaire suitable to the local context and focus on the risk behaviors and beliefs associated with NCDs, the researchers discussed and agreed to extract the items from STEPS.

The interviews were conducted by the surveyors from the Bridge of Community Development Foundation, a non-government organization registered by the Bangladesh government. The surveyor randomly selected 100 candidates from each union mentioned above. A total of 600 candidates were selected from six unions. The convenience sampling method was adapted for each union. After obtaining verbal consent, the questionnaire survey was administered to each participant. If the participants had difficulty with reading and writing, the surveyors assisted them in completing the questionnaire. Subsequently, blood pressure, sugar level in the blood, height, and weight were measured by the surveyors. The sugar level was measured using a glucometer by surveyors in the field. The participants were informed about these measurements immediately.

### 2.3. Outcomes

To identify the different groups for risk behavior and belief patterns associated with NCDs, the questionnaire survey items retrieved from STEPS were used. The survey items consisted of three categories: risk behavior (six items), beliefs (three items), and improvement behavior (eight items). Risk behavior included smoking habits, eating less fruit, eating less vegetables, adding salt to food, eating processed food high in salt, and less physical activity. Beliefs included the importance of reducing salt, the association between salt and health problems, and the association between sugar or fat and health problems. Improvement behavior refers to the behavior associated with improving the NCDs within the last 3 months; we asked for improvement behavior using the following question, “in the past 3 months, did you do any of the following?”, with the following possible responses: reduce salt in your diet, eat at least five servings of fruit and/or vegetables each day, reduce fat in your diet, start or do more physical activity, maintain a healthy body weight or lose weight, reduce sugary beverages in your diet, never read or watched TV programs on health, and never asked medical staff for information about health. Participants who had been diagnosed with hypertension previously by medical doctors or had a systolic blood pressure of over 140 or a diastolic blood pressure of over 90 were defined as participants with hypertension. Participants who had been diagnosed previously by medical doctors with diabetes or had a blood sugar level over 13.0 mmol/L were defined as participants with diabetes. Body mass index (BMI) was calculated using height and weight measurements.

### 2.4. Statistical Analysis

The participants’ basic characteristics were summarized by each age group. Next, a logistic regression analysis was performed with each comorbidity (hypertension, diabetes, and BMI ≥ 25) as dependent variables. The independent variables for each comorbidity model were selected using the Akaike information criterion (AIC) and the Bayesian information criterion (BIC), as the number of variables suitable for inclusion was limited owing to the small sample size. Further, to identify the different groups based on risk behavior and belief patterns associated with NCDs, a log-likelihood latent class analysis was conducted. Risk behaviors and beliefs associated with NCDs were used for analysis as binary variables. Entropy, AIC, BIC, and the proportion of participants in each group were used for clustering. The participants’ basic characteristics by cluster group, as the different groups based on risk behavior and belief patterns associated with NCDs, were summarized. Then, a multinomial regression analysis was performed to identify the factor associated with each cluster group. The demographics model included risk behavior and belief group as the dependent variables, and included sex, age, education years, income, and BMI as independent variables. The comorbidity model included risk behavior and belief group as the dependent variables, and included sex, age, education years, daily medicine taken, presence of hypertension, and presence of diabetes as independent variables. All analyses were performed using STATA IC15 (Lightstone, San Antonio, TX, USA, version 15) and Mplus version 8 (Muthén & Muthén, Los Angeles, CA, USA).

## 3. Results

A total of 600 participants were included in the final sample for analysis. In the sample, the median age was 45 (IQR; 39–56), 280 (46.7%) were women, and 359 (59.8%) could read and write. The major occupation was agriculture and fishing, and 396 (66.0%) participants’ monthly incomes were under 200 USD. A total of 152 (25.2%) participants took medicine daily, 231 (38.5%) had hypertension, 87 (14.5%) had diabetes, and 209 (34.8%) had a BMI of 25 or more. The median ages of male and female participants were 50 (IQR; 40–58) and 45 (IQR; 38–55), respectively. Hypertension and diabetes were found in 112 (35.0%) and 27 (8.4%) male participants, respectively, and hypertension and diabetes were found in 119 (42.5%) and 45 (16.1%) female participants, respectively. The proportion of participants with hypertension was 18.2% in the under-40 age group and 61.9% in the over-59 age group. The proportion of participants with diabetes was 9.7% in the under-40 age group and 23.7% in the over-59 age group (Table 1). Among those without hypertension, 109 (29.5%) participants had a BMI of 25 or more, while 100 (43.3%) participants with hypertension had a BMI of 25 or more (Table S1).

The logistic regression analysis with hypertension as a dependent variable indicated that female gender, age, more education years, and high BMI were associated with hypertension. The logistic regression analysis with diabetes as a dependent variable indicated that hypertension was the only variable associated with diabetes. The logistic regression analysis with overweight and obesity (BMI ≥ 25) as a dependent variable showed that female gender and a high household income were associated with being overweight and obesity. Notably, the population characteristics of participants with hypertension were distinct in contrast to diabetes and overweight, for which these characteristics were not distinct (Table 2).

**Figure 1:**
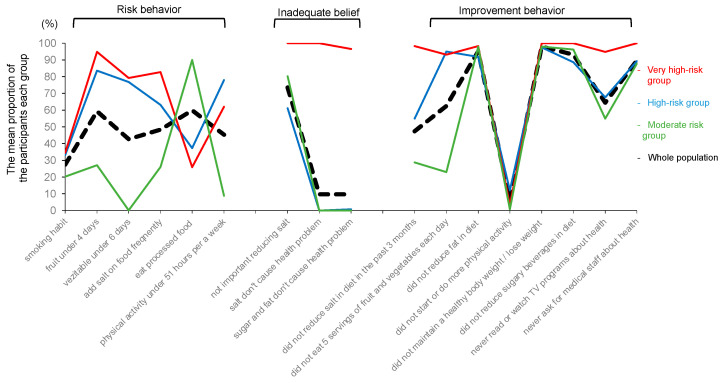
Probabilities of behaviors and beliefs associated with NCDs for the three cluster groups (N = 600). (1) Very high-risk group (*n* = 58); high-risk behaviors, wrong beliefs, and less improvement behaviors; (2) high-risk group (*n* = 270); high-risk behaviors, right beliefs, and moderate improvement behaviors; (3) moderate-risk group (*n* = 272); low-risk behaviors, right beliefs, and moderate improvement behaviors.

The researchers determined the number of classes of risk behavior and belief patterns associated with NCDs by entropy, AIC, BIC, and the proportion of individuals in each class (Table 3). Entropy was 0.887 for class 2, 0.934 for class 3, 0.907 for class 4, and 0.899 for class 5. Considering other factors, three cluster groups were defined. The mean proportions of the participants in each cluster group and the whole population are shown in Figure 1. Three cluster groups were named as follows: (1) very high-risk group (*n* = 58), which was characterized by high-risk behaviors, wrong beliefs, and less improvement behaviors; (2) high-risk group (*n* = 270), which was characterized by high-risk behaviors, right beliefs, and moderate improvement behaviors; and (3) moderate-risk group (*n* = 272), which was characterized by low-risk behaviors, right beliefs, and moderate improvement behaviors.

The figure shows the characteristics of the three cluster groups based on participant responses to 16 variables.

The median age was highest and the median education year was lowest in the very high-risk group, and 81% of the participants in the very high-risk group had a monthly income of under 200 USD (Table S2).

Multinomial regression analyses were performed to identify the factor that influenced the differences between cluster groups. The very high-risk group was significantly associated with female gender, high age, low educational years, and absence of daily medication compared to the moderate-risk group. The high-risk group was associated with female gender, high age, low educational years, and absence of hypertension compared to the moderate-risk group (Table 4).

In addition, we present the relationship between the processed food intake and the presence of obesity and diabetes in Table S3. The proportions of overweight and obesity were higher among the residents who sometimes ate processed food high in salt (Table S3).

## 4. Discussion

The patterns of risk behaviors and beliefs associated with NCDs had not been identified in rural areas of developing countries in previous studies. We determined different groups of risk behaviors and beliefs associated with NCDs and the factors that influence these cluster groups in rural Bangladesh, where NCDs are a major concern.

Risk behaviors and beliefs associated with NCDs among rural residents in Bangladesh were classified into three groups: (1) very high-risk group, characterized by high-risk behavior, wrong belief, and less improvement behavior; (2) high-risk group, characterized by high-risk behavior, right belief, and moderate improvement behavior; and (3) moderate-risk group, characterized by low-risk behavior, right belief, and moderate improvement behavior. Previous studies on hypertension patients reported that the high-risk group for hypertension consists of individuals who consume too much salt and oil [27]. Hence, interventional actions should be developed based on the characteristics of each group to more effectively reduce NCDs: modify beliefs for the very high-risk group, modify risk behaviors for the high-risk group, and modify improvement behaviors for the moderate-risk group.

The proportion of patients with hypertension was very high and distinctive in the rural areas of Bangladesh. Specifically, it was 18.2% in the under-40 age group and 61.9% in the over-59 age group. The results of the multivariate analysis showed that hypertension was more common among older adults, women, the group with more education years, and those with a BMI of 25 or over. Several causes of these high proportions of hypertension in LICs and MICs were reported, for instance, malnutrition during the fetal stage [28]. Moreover, dietary habit problems such as the excessive consumption of snacks and salty foods and the exclusion of vegetables from the diet might be severe in areas where this study was conducted. Unmet healthcare needs were severe in these areas, and the healthcare providers could not manage NCD patients along with maternal and child health and infectious diseases. The intervention strategies by non-medical healthcare staff for NCD patients through preventive medicine and education for dietary habit improvement should be immediately implemented in these areas, where medical resources and household incomes are low.

The classification of the very high-risk group and the high-risk group based on risk behaviors and beliefs was conducted considering the participants’ social demographic characteristics. Factors associated with the very high-risk and high-risk groups were high age, female gender, and shorter educational years. A previous interventional study that used an educational checklist for pesticide protection in Bangladesh showed that the effect of educational intervention was smaller in groups consisting of individuals who were older and had shorter educational years [29]. The proportions of aging individuals, females, and those with short education years in the very high-risk and high-risk groups were quite high. Thus, more accommodating support should be considered for these groups when applying educational interventions.

Among the participants, hypertension was more common among women than men. The proportion of hypertension was 42.5 among female rural residents and 35.0% among male rural residents. The proportion of female participants with hypertension was 20.0% in the under-40 age group, 45.5% in the 40–59 age group, and 77.8% in the over-59 age group. In contrast, a previous study reported that hypertension was more prevalent among men in younger age groups, such as those under the age of 60 [30]. In this study, a higher prevalence of hypertension was noted among women in rural Bangladesh probably because, compared to the men, the majority of women in this region were housewives who did not go out into the community often and spent most of their time indoors and, consequently, did not have sufficient access to knowledge about NCDs. Moreover, they were in charge of preparing daily meals for the entire household. Thus, providing these women with knowledge about NCDs, especially about proper dietary habits, is vital to promoting healthy behaviors of all household members.

Several limitations should be considered while generalizing the results of this study. First, the sample size for the latent class analysis was small; however, it should be noted that this size was considered permissible in previous studies [31]. Second, blood sugar level was measured as casual blood glucose level. Fasting blood glucose level and other blood tests should be considered to intensify the proportion of patients with diabetes more precisely. Third, the mean proportion of participants for risk behaviors, beliefs, and improvement behaviors had biased distributions, especially for several items of beliefs and improvement behaviors. Further data on risk behaviors and beliefs associated with NCDs more suitable for latent class analysis should be obtained. Fourth, we could not consider the sample size of each union for sampling methods. Fifth, hypertension and diabetes proportions were lower among very the high-risk group, because the patients who have hypertension or diabetes have already changed their lifestyle to improve their disease. For discussion of how the cluster of NCD risk behavior affects the risk of having NCD, further cohort studies among heathy people were required. Sixth, we did not measure the examination item for metabolic syndrome. Waist measurement, triglyceride, and HDL cholesterol should be measured to identify metabolic syndrome. Seventh, we did not identify work-related physical activity; we only examined all physical activity. Despite these limitations, this study was the first to identify cluster groups of risk behaviors and beliefs associated with NCDs through a latent class analysis in the community of rural Bangladesh, which experienced a rapid increase in NCD cases.

## 5. Conclusions

In this study, the very high-risk group and the high-risk group of risk behaviors and beliefs related to NCDs were influenced by high age, female gender, and shorter education years. Further, the proportion of participants with hypertension was very high in the rural areas of Bangladesh, especially among women. Thus, educational interventions for improving the risk behaviors and beliefs associated with NCDs should be immediately implemented in these rural areas where medical resources are limited.

## Figures and Tables

**Table 1:** Characteristics of participants by age group (N = 600).

	Under 40 Years Old (*n* = 154)	40–59 Years Old (*n* = 328)	Over 59 Years Old (*n* = 118)
Sex (female)	90 (58.4)	145 (44.2)	45 (38.1)
Education years (median [IQR])	9 [5–11]	5 [0–10]	0 [0–8]
Income			
Under 200 USD	89 (57.8)	214 (65.2)	93 (78.8)
200–399 USD	57 (37.0)	102 (31.1)	23 (19.5)
400–599 USD	8 (5.2)	10 (3.1)	1 (0.9)
600–800 USD	0 (0.0)	2 (0.6)	1 (0.9)
Occupation			
Students	7 (4.6)	0 (0.0)	0 (0.0)
Service work with employed	1 (0.7)	3 (0.9)	1 (0.9)
Agriculture and fishing	28 (18.2)	116 (35.4)	56 (47.5)
Own business	33 (21.4)	75 (22.9)	20 (17.0)
Housewife	61 (39.6)	110 (33.5)	40 (33.9)
Employed	24 (15.6)	24 (7.3)	1 (0.9)
Number of household members (median [IQR])	4 [3–5]	4 [4–6]	5.5 [3–7]
Medicine taken daily (yes)	10 (6.5)	82 (25.0)	59 (50.0)
Previous hypertension diagnosis	5 (3.3)	66 (20.1)	45 (38.1)
Previous diabetes diagnosis	7 (4.6)	34 (10.4)	20 (17.0)
Previous dyslipidemia diagnosis	0 (0.0)	4 (1.2)	4 (3.4)
BMI			
Normal (18.5–24.9)	81 (52.6)	189 (57.6)	69 (58.5)
Underweight (<18.5)	9 (5.8)	27 (8.2)	16 (13.6)
Overweight (25–29.9)	48 (31.2)	88 (26.8)	30 (25.4)
Obesity (>30)	16 (10.4)	24 (7.3)	3 (2.5)
Hypertension (found during survey or diagnosed previously)	28 (18.2)	130 (39.6)	73 (61.9)
Diabetes (found during survey or diagnosed previously)	15 (9.7)	44 (13.4)	28 (23.7)

Data are *n* (%) unless specified otherwise. IQR = interquartile range.

**Table 2:** Logistic regression analysis with each comorbidity as the dependent variable (N = 600).

	Dependent Variable: Hypertension		Dependent Variable: Diabetes		Dependent Variable: Overweight and Obesity	
	OR [95% CI]	*p*-Value	OR [95% CI]	*p*-Value	OR [95% CI]	*p*-Value
Sex (female)	1.72 [1.18–2.53]	0.005	1.27 [0.79–2.04]	0.33	2.30 [1.59–3.32]	<0.001
Age (base: under 40 years old)						
40–59	4.31 [2.56–7.25]	<0.001	1.56 [0.83–2.93]	0.16	0.97 [0.63–1.49]	0.88
Over 59	13.0 [6.89–24.59]	<0.001	3.34 [1.66–6.72]	0.001	0.84 [0.48–1.48]	0.55
Education (years)	1.04 [1.00–1.09]	0.046			1.04 [1.00–1.48]	0.058
BMI (base: normal and underweight)						
Overweight (25–29.9)	1.69 [1.13–2.55]	0.011	2.00 [1.21–3.29]	0.007		
Obesity (>30)	4.17 [2.06–8.43]	<0.001	1.15 [0.69–4.10]	0.25		
Household income (over 200 USD)					1.52 [1.01–2.27]	0.042

OR = odds ratio, CI = confidential interval.

**Table 3:** Risk behavior, beliefs, and improvement behavior associated with non-communicable diseases.

	Answered Yes (*n* (%))
Risk behavior	
Smoking habit	165 (27.5)
Eating fruit less than 4 days in a week	356 (59.3)
Eating vegetables less than 6 days in a week	256 (42.7)
Adding salt to food frequently	290 (48.3)
Eating processed food high in salt	359 (59.8)
Physical activity under 51 h per week	272 (45.3)
Beliefs	
Reducing salt intake is not important.	440 (73.3)
Salt does not cause health problems.	58 (9.7)
Sugar and fat do not cause health problems.	58 (9.7)
Improvement behavior within last 3 months	
Did not reduce salt in diet in the past 3 months	284 (47.3)
Did not eat five servings of fruits and vegetables each day	375 (62.5)
Did not reduce fat in diet	571 (95.2)
Did not increase the time of physical activity	37 (6.2)
Did not maintain a healthy body weight or lose weight	588 (98.0)
Did not reduce the intake of sugary beverages	559 (93.2)
Never read or watched TV programs on health	387 (64.5)
Never asked medical staff for information about health	538 (89.7)

**Table 4:** Multinomial regression analysis with each risk behavior and belief group as the dependent variable (N = 600).

	Demographics Model		Comorbidity Model	
	RRR [95% CI]	*p* Value	RRR [95% CI]	*p* Value
Very high-risk group (vs. moderate-risk group)				
Sex (base: male)	1.71 [0.90–3.23]	0.099	2.00 [1.04–3.83]	0.036
Age (years)	1.06 [1.03–1.09]	<0.001	1.09 [1.06–1.13]	<0.001
Education (years)	0.81 [0.74–0.89]	<0.001	0.82 [0.75–0.89]	<0.001
Income	1.24 [0.56–2.75]	0.61		
BMI				
Underweight (<18.5)	2.18 [0.92–5.18]	0.076		
Overweight (>25)	0.50 [0.22–1.09]	0.082		
Taken medicine daily (base: none)			0.24 [0.08–0.75]	0.013
Hypertension			0.63 [0.29–1.38]	0.25
Diabetes			0.60 [0.15–2.36]	0.46
High-risk group (vs. moderate-risk group)				
Sex (base: male)	1.59 [1.09–2.32]	0.016	1.61 [1.11–2.34]	0.013
Age (years)	1.04 [1.02–1.05]	<0.001	1.04 [1.02–1.06]	<0.001
Education (years)	0.94 [0.89–0.98]	0.004	0.95 [0.91–0.99]	0.021
Income	1.31 [0.87–1.99]	0.20		
BMI				
Underweight (<18.5)	0.87 [0.43–1.75]	0.70		
Overweight (>25)	1.07 [0.73–1.56]	0.74		
Taken medicine daily (base: none)			1.25 [0.73–2.16]	0.42
Hypertension			0.63 [0.41–0.99]	0.045
Diabetes			1.11 [0.64–1.93]	0.72

RRR = relative risk ratio

## Data Availability

The datasets generated and/or analyzed during the current study are not publicly available, as they include participants’ personal information. However, they are available from the corresponding author on reasonable request.

## Supplementary Materials

The following supporting information can be downloaded at https://www.mdpi.com/article/10.3390/healthcare11162279/s1, Figure S1: Map of the study site (Narail district) in Bangladesh; Table S1: Participant characteristics by presence of hypertension; Table S2: Participant characteristics by risk behaviors and belief groups. Table S3: Association between eating processed food and diabetes, BMI.
