# Supplementary material for: Examination of Cluster Groups of Risk Behaviors and Beliefs Associated with Non-Communicable Diseases with Latent Class Analysis: A Cross-Sectional Study in Rural Bangladesh
Source: Healthcare (Basel). 2023 Aug 12;11(16):2279. doi: 10.3390/healthcare11162279 (PMC10454554; doi:10.3390/healthcare11162279)
Supplement: Supplementary file 1 [file healthcare-11-02279-s001.zip › healthcare-2477493-supplementary.pdf]

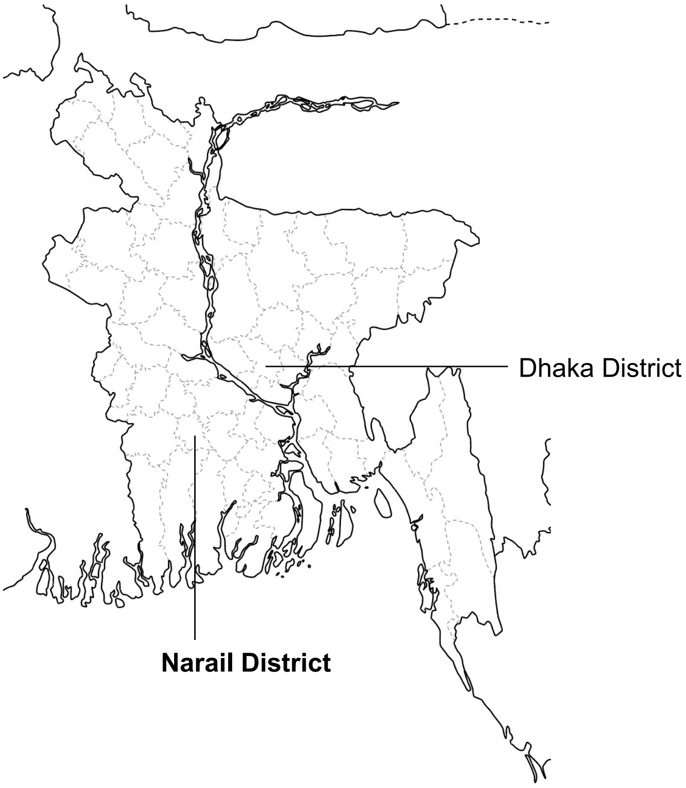

Figure S1. The map of the study sight (Narail district) in Bangladesh.

Table S1. Participant characteristics by the presence of hypertension

	No hypertension (n = 369)	Hypertension (n = 231)
Age (median [IQR])	42 [37–52]	55 [45–60]
Sex female	161 (43.6)	119 (51.5)
BMI		
18.5–25	223 (60.4)	116 (50.2)
≦18.5	37 (10.0)	15 (6.5)
25–30	92 (24.9)	74 (32.0)
≧30	17 (4.6)	26 (11.3)
Income		
Under 200 USD	244 (66.1)	152 (65.8)
200–400 USD	110 (29.8)	72 (31.2)
400–600 USD	13 (3.5)	6 (2.6)
600–800 USD	2 (0.5)	1 (0.4)
Smoking habit (yes)	99 (26.8)	66 (28.6)
Frequency of adding salt to food		
Always	179 (48.5)	81 (35.1)
Often	13 (3.5)	17 (7.4)
Sometimes	55 (14.9)	39 (16.9)
Rarely	59 (16.0)	45 (19.5)
Never	63 (17.1)	49 (21.2)
Frequency of eating processed food high in salt		
Always	0 (0.0)	0 (0.0)
Often	0 (0.0)	0 (0.0)
Sometimes	20 (5.4)	7 (3.0)
Rarely	202 (54.7)	130 (56.3)
Never	147 (39.8)	94 (40.7)
Belief about lowering salt intake in diet		
Very important	98 (26.6)	61 (26.4)
Somewhat important	213 (57.7)	143 (61.9)
Not at all important	58 (15.7)	26 (11.3)
Too much salt in diet could cause health problems.		
Yes	329 (89.2)	212 (91.8)

Data are n (%) unless specified otherwise. IQR = interquartile range

Table S2. Participant characteristics by risk behaviors and belief groups

	Very high-risk group (n = 58)	High-risk group (n = 270)	Moderate-risk group (n = 272)
Age (median [IQR])	54.5 [45–60]	48.5 [40–56]	42 [33.5–55]
Sex (female)	29 (50.0)	137 (50.7)	114 (41.9)
Education years (median [IQR])	0 [0–5]	5 [0–9]	8 [0–10]
Income			
Under 200 USD	47 (81.0)	179 (66.3)	170 (62.5)
200–400 USD	11 (19.0)	88 (32.6)	83 (30.5)
400–600 USD	0 (0.0)	3 (1.1)	16 (5.9)
600–800 USD	0 (0.0)	0 (0.0)	3 (1.1)
Occupation			
Student	0 (0.0)	0 (0.0)	7 (2.6)
Service work	0 (0.0)	2 (0.7)	3 (1.1)
Agriculture and fishing	22 (37.9)	99 (36.7)	79 (29.0)
Own business	6 (10.3)	54 (20.0)	68 (25.0)
Housewife	28 (48.3)	101 (37.4)	82 (30.2)
Employed	2 (3.5)	14 (5.2)	33 (12.1)
Number of household members (median [IQR])	4.5 [3–6]	5 [4–6]	4 [4–6]
Medicine taken	7 (12.1)	85 (31.5)	59 (21.7)
Previous hypertension diagnosis	6 (10.3)	60 (22.2)	50 (18.4)
Previous diabetes diagnosis	2 (3.5)	35 (13.0)	24 (8.8)
Previous dyslipidemia diagnosis	1 (1.7)	3 (1.1)	4 (1.5)
BMI			
Normal (18.5–25)	35 (60.3)	150 (55.6)	154 (56.6)
Underweight (≦18.5)	13 (22.4)	19 (7.0)	20 (7.4)
Overweight (25–29)	8 (13.8)	81 (30.0)	77 (28.3)
Obesity (30≦)	2 (3.5)	20 (7.4)	21 (7.7)
Hypertension (found during the survey or diagnosed previously)	18 (31.0)	108 (40.0)	105 (38.6)
Diabetes (found during the survey or diagnosed previously)	1 (1.7)	30 (11.1)	28 (10.3)

Data are n (%) unless specified otherwise. IQR = interquartile range

Table S3. **Association between eating processed food and diabetes, BMI**

	Frequency of eating processed food high in salt		
	Sometimes	Rarely	Never
Diabetes (found during survey or diagnosed previously)			
No	25 (4.9)	286 (55.8)	202 (39.4)
Yes	2 (2.3)	46 (52.9)	39 (44.8)
BMI			
Normal (18.5–24.9)	11 (3.2)	189 (55.8)	139 (41.0)
Underweight (< 18.5)	1 (1.9)	31 (59.6)	20 (38.5)
Overweight (25–29.9)	13 (7.8)	86 (51.8)	67 (40.4)
Obesity (> 30)	2 (4.7)	26 (60.5)	15 (34.9)

Data are n (%) unless specified otherwise.
